# Three-Stage Treatment of Transcalcaneal Talonavicular Fracture Dislocation; A Case Report and Literature Review 

**DOI:** 10.29252/beat-070411

**Published:** 2019-10

**Authors:** Amir Reza Vosoughi, Mohammad Jafar Emami

**Affiliations:** 1 *Bone and Joint Diseases Research Center, Department of Orthopedics Surgery, Shiraz University of Medical Sciences, Shiraz, Iran*

**Keywords:** Calcaneus, Talus, Transcalcaneal talonavicular fracture dislocation, Subtalar, Calcaneocuboid

## Abstract

Transcalcaneal talonavicular fracture dislocation is an extremely rare debilitating injury with high complication rates. The present case report demonstrates highly comminuted joint-depressed fracture of left calcaneus treated with primary subtalar arthrodesis following reduction and fixation of the calcaneus. The right ankle sustained a highly comminuted fracture of calcaneal body with completely-destroyed posterior facet, fracture dislocation of the calcaneocuboid joint, dorsally dislocated talonavicular joint, fracture of anterior of tibial plafond, and subluxation of the tibiotalar joint. At first, talonavicular joint was reduced and fixed using a plate followed by reduction of calcaneus and arthrodesis of subtalar and calcaneocuboid joints. The plate of talonavicular joint was removed after 70 days. Logical approach to this injury can lead to an acceptable function.

## Introduction

Fracture of the calcaneus is seen in about 0.4% to 2.0 % of all fractures presented to the emergency departments. Displaced intraarticular calcaneal fracture is the most common form [1, 2]. One of the most severe types of hindfoot injury is transcalcaneal talonavicular fracture dislocation. This extremely rare entity is a combination of highly comminuted calcaneal fracture in addition to the dorsal dislocation or subluxation of the talonavicular joint. Instability of the calcaneocuboid joint with comminuted intraarticular fracture is usual. Talus may be fractured or not. Transcalcaneal talonavicular fracture dislocation usually is the result of a high-energy mechanism of injury with associated fractures in the lower limb and the spine [3]. To the best of our knowledge based on the literature review, about 20 cases have been reported with debilitating outcomes such as amputation in some of them [3-10]. In the present case, we describe a case of right-sided closed transcalcaneal talonavicular fracture dislocation treated successfully in three stages.

## Case Report

**Fig. 1:**
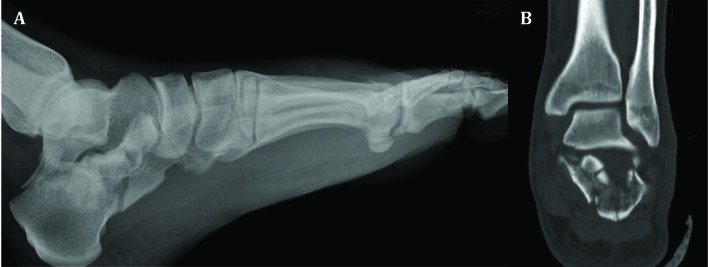
(A) Lateral radiograph and (B) coronal CT section of left ankle showing comminuted joint-depressed fracture of the calcaneus (Sanders type IV).

**Fig. 2:**
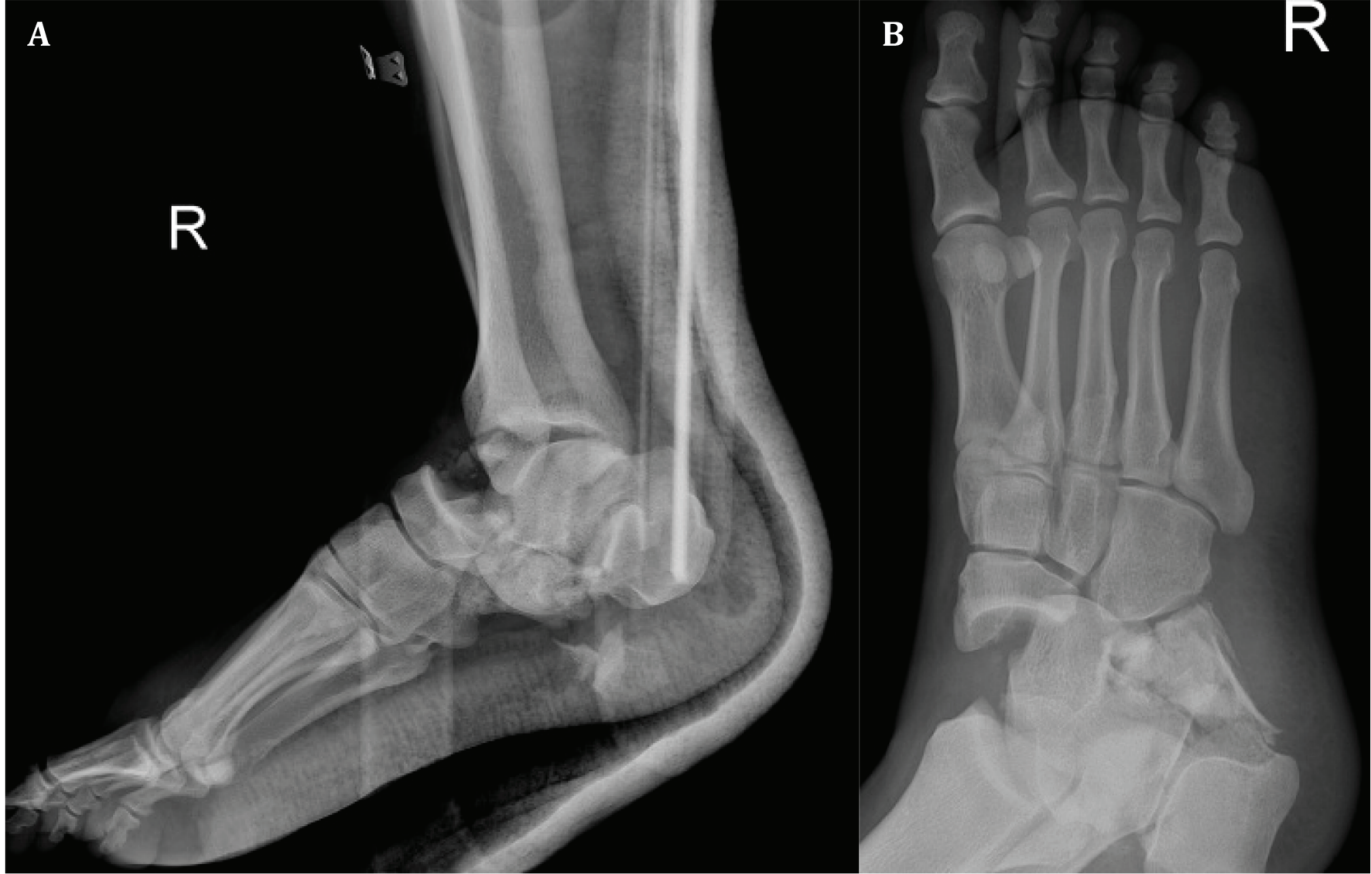
(A) Lateral of right ankle and (B) oblique of right foot radiographs revealing dislocated talonavicular joint with displaced calcaneal fracture and multiple fragments in the plantar area

**Fig. 3:**
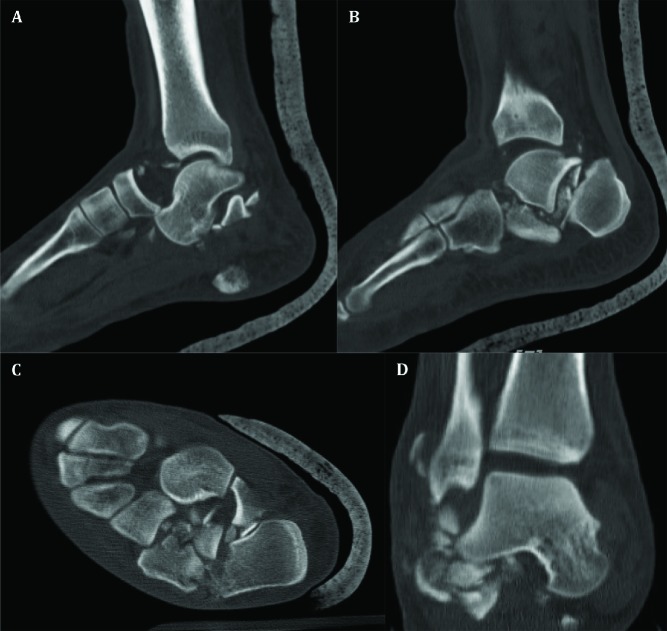
CT-scan of right ankle in sagittal (A, B), axial (C) and coronal (D) views demonstrating highly-comminuted fracture of the calcaneus, dislocated talonavicular joint, subluxation of the tibiotalar joint with multiple free fragments

A healthy 27-year-old car-driver man was consulted for bilateral painful ankle fractures, five days after a motor vehicle collision in a highway. He had undergone spinal surgery because of burst fracture of the fifth lumbar vertebra with incomplete spinal cord injury. Severe swelling, bullae, ecchymosis, and deformities without any laceration indicative of open fracture were obvious around both feet and ankles extended proximally especially in the right side. The neurovascular status of both feet was intact. Left ankle plain radiograph and computed tomography (CT) scan revealed comminuted joint-depressed fracture of the calcaneus classified as Sanders type IV (Figure 1). Right ankle x-ray (Figure 2) and CT scan (Figure 3) showed highly-comminuted fracture of the calcaneal body with completely-destroyed posterior facet, fracture dislocation of the calcaneocuboid joint, dorsally dislocated talonavicular joint, fracture of anterior lip of tibial plafond, and subluxation of the tibiotalar joint.

**Fig. 4:**
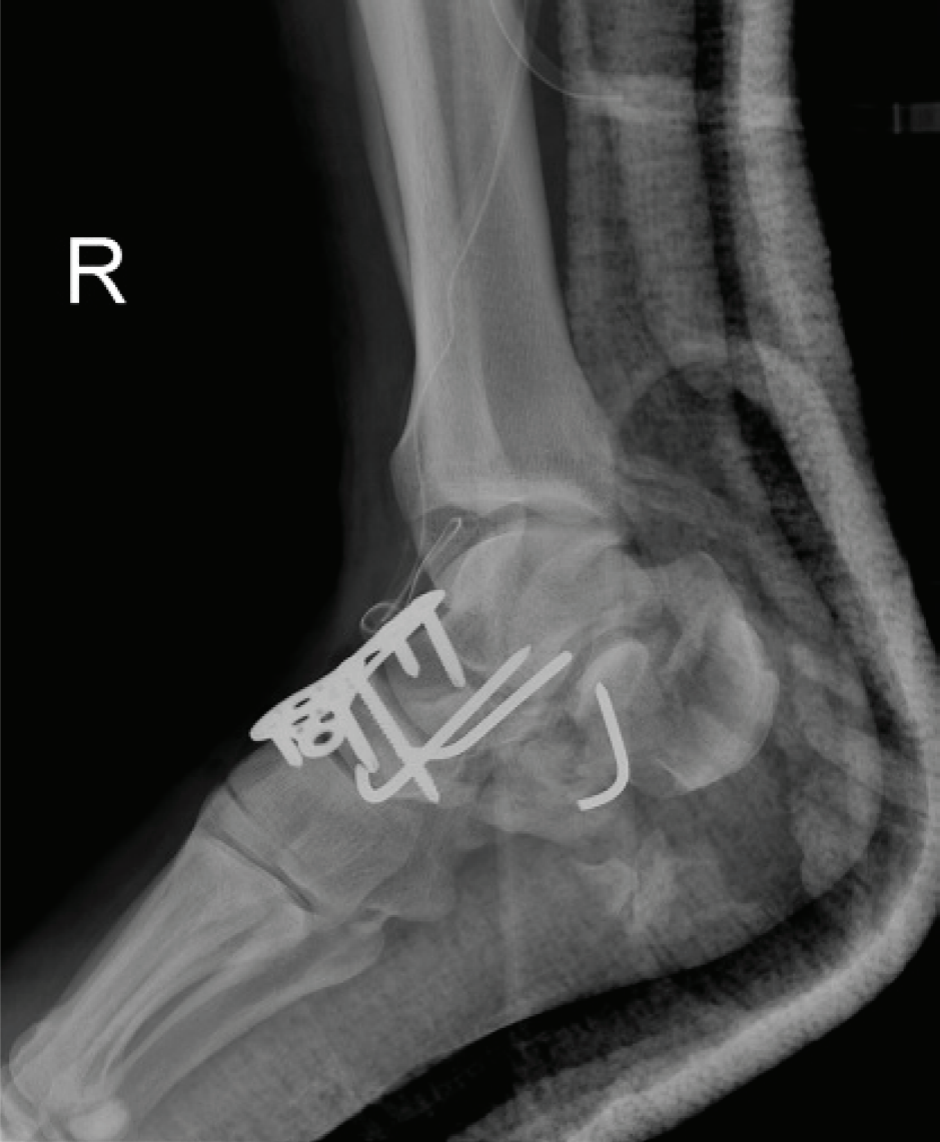
Lateral radiograph after the first surgery

**Fig. 5:**
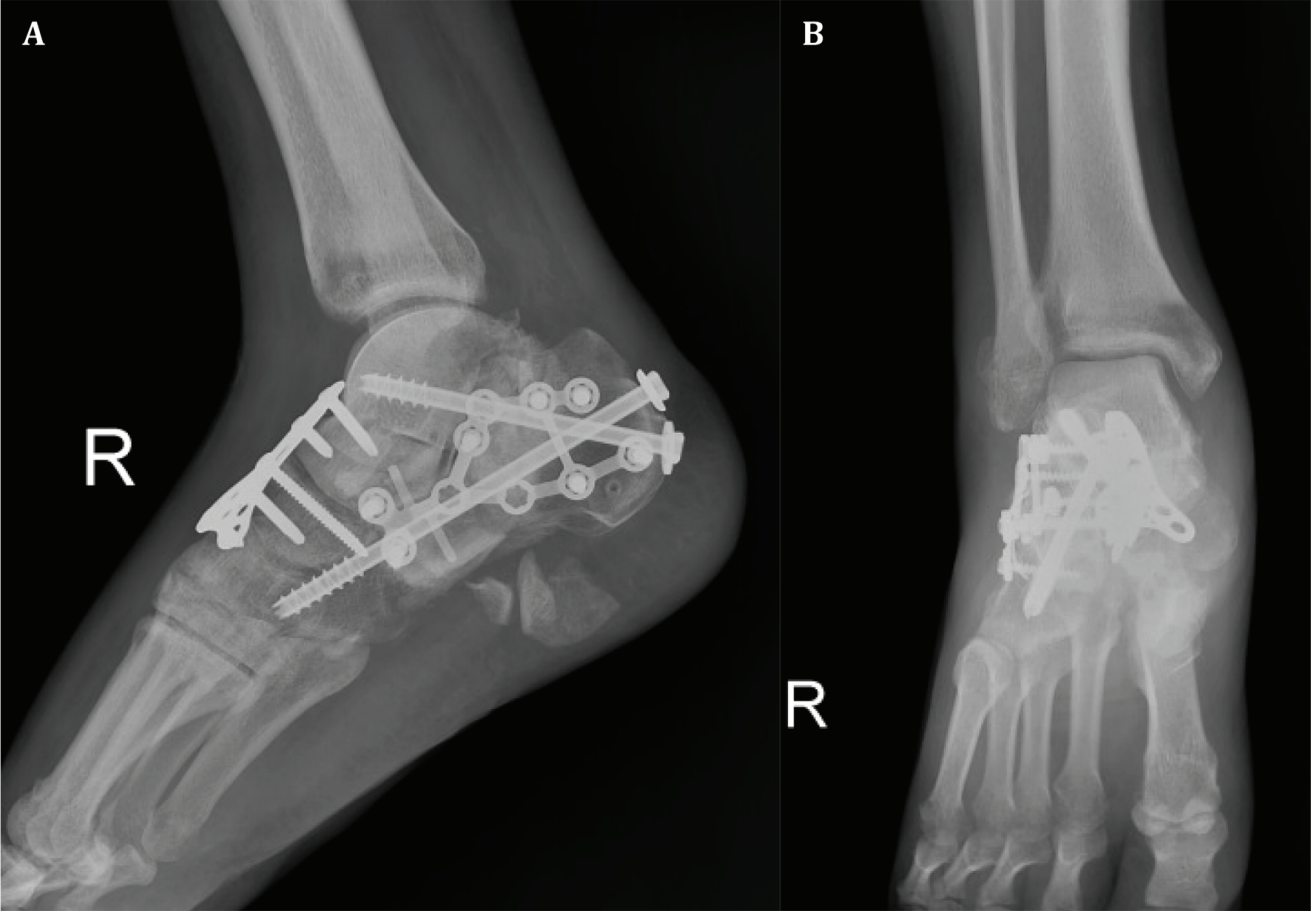
(A) Lateral and (B) anteroposterior radiographs of right ankle after the second surgery

**Fig. 6:**
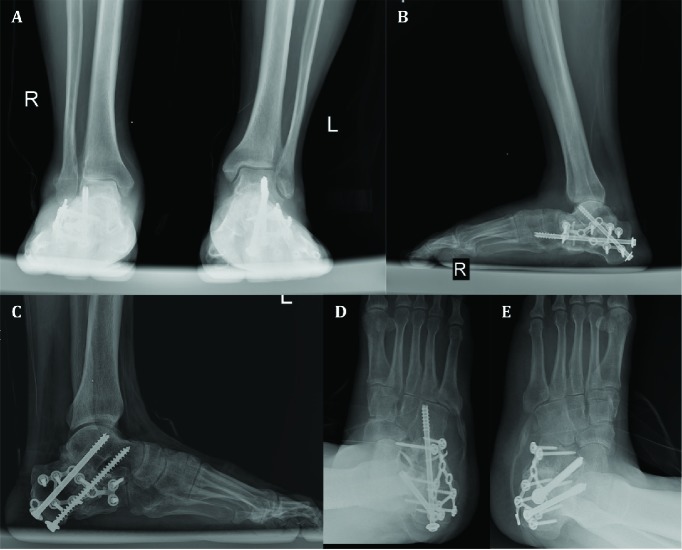
At 14-month postoperative follow-up, anteroposterior ankles (A), lateral ankles (B, C), and oblique feet (D, E) radiographs demonstrating bony union

**Fig. 7:**
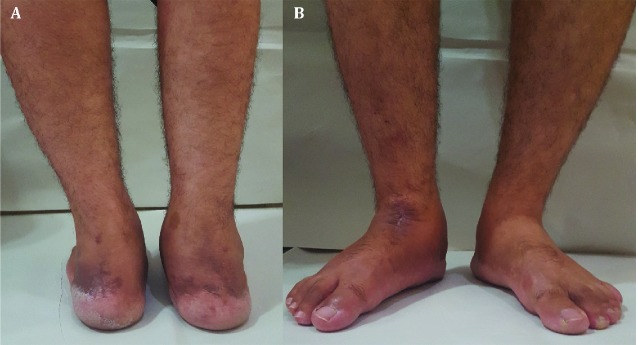
Photographs of feet and ankles (A, B), 14 months after the surgery showing normal alignment, smaller size of right calf muscles but without any wound problem or infection

In the first stage, 10 days following the trauma, after inflation of the tourniquet, through dorsal approach to the right ankle and talonavicular joints, the joints were opened. Soft tissues and multiple osteocartilaginous fragments from anterior part of the tibial plafond were removed. The talonavicular joint was reduced and fixed using two percutaneous k-wires and a T-plate. Moreover, calcaneal fracture was manipulated closely to achieve better alignment and fixed using a percutaneous k-wire (Figure 4). Short leg non-weight bearing slab was applied. In that stage after changing the position of the patient to the lateral decubitus, left calcaneal fracture was reduced and fixed using a calcaneal plate followed by primary subtalar arthrodesis via extensile lateral approach. Bone allograft was used to fill the defective parts of the calcaneus. Short leg non-weight bearing cast was enrolled and changed to slab after 6 weeks. 

In the second stage, 10 days later, when swelling and bullae had subsided, after removal of all k-wires, open reduction and internal fixation of calcaneus via extensile lateral approach was done. The massive bone defect in the calcaneus and some parts of the calcaneocuboid joint was replaced by a big structural autograft bone harvested from the iliac crest followed by subtalar and calcaneocuboid arthrodesis (Figure 5). Postoperatively, a non-weight bearing short leg cast was applied for 6 weeks. This was converted to a short leg non-weight bearing slab in order to start range of motion exercises.

In the third stage, 70 days after the first operation, T-plate which had been inserted on dorsal of talonavicular joint was removed beside resecting multiple big bone fragments in the plantar of calcaneus. After 2 weeks in a short leg non-weight bearing slab, partial weight bearing was started and advanced to full.

At the final follow-up, 14 months following the trauma, he can walk without any aid but with obvious gait abnormality mainly because of weakness of the first sacral nerve root after L5 burst fracture. On examination, passive range of motion of the right ankle joint was 15 degrees’ plantarflexion to 10 degrees’ dorsiflexion and the left side had the arch from 40 degrees’ plantarflexion to 15 degrees’ dorsiflexion. Visual analogue score was 4 for the right side and 8 for the left one. American orthopaedic foot and ankle society ankle-hindfoot scale for the right side was 60 (pain: 20, Function: 30, alignment: 10) and for the left one, it was 74 (pain: 30, Function: 34, alignment: 10). It should be mentioned that bilateral involvement may have some false effects on the function section of American orthopaedic foot and ankle society ankle-hindfoot scale. Complete bony union without broken device or obvious arthritic changes in the adjacent joints were seen (Figures 6 and 7). It should be mentioned that informed consent for publication of this case report has been obtained from the patient.

## Discussion

Transcalcaneal talonavicular fracture dislocation is a potentially limb-threatening injury with very poor prognosis. The hallmark of these injuries, as described by Ricci *et al*., [3], is plantar dislocation of talar head from the talonavicular joint with crushing of the anterior part of the calcaneus and destruction of the calcaneocuboid joint. Fracture of the talar head, the navicular, the tibial plafond, and the tibiotalar subluxation or dislocation could be seen [3, 6, 9]. Associated laceration of important structures such as neurovascular bundles and tendons is common (3 out of 9 limbs described by Ricci *et al*., [3].

In confronting to these severe injuries, preservation of the limb should be the first goal. Below-knee or Syme amputation might be expected in 33% to 60% of cases (3 out of 5 reported by Coltart [4] and 3 out of 9 by Ricci *et al*., [3]. Based on Ricci *et al*., [3], two out of three amputations were done in the closed wound injuries. It could be concluded that rational management of severe closed soft tissue injuries in transcalcaneal talonavicular fracture dislocation is a necessity. The treatment of the present case was planned in a 70-day period in three different stages in order to permit the soft tissues to resolve their extensive damages.

Based on the high severity of this injury, open transcalcaneal talonavicular fracture dislocation is often seen. The most reported cases had medial [3, 8] or plantar lacerations [10]. Although closed transcalcaneal talonavicular fracture dislocation following high-energy trauma was reported by Ricci *et al*., [3] similar to our presented case, they could be seen in low-energy mechanism such as a closed transcalcaneal fracture with talonavicular subluxation in an 86-year-old passenger female following a car to wall impact collision [7] and a 71-year-old female following a fall from a step [9]. 

Among all reported cases in the literature, a case reported by Ricci *et al*., [3] was similar to ours. They described a patient of bilateral closed transcalcaneal talonavicular fracture dislocation with lumbar spine fracture. The final result was below knee amputation of the right side after four operations and surgical wound infection of the left one after 3 surgeries. At first, they used external fixator to maintain reduction of the talus in the talonavicular joint. Subtalar and calcaneocuboid joint arthrodesis was done after 2 weeks. Our case had closed left-sided Sanders type IV calcaneal fracture and closed right-sided transcalcaneal talonavicular fracture dislocation in association with lumbar vertebra fracture. It was decided to use a plate in addition to k-wires in order to keep it for a long time without increasing the risk of infection, one of the common reported complications [3]. 

Closed transcalcaneal talonavicular fracture dislocation should be approached cautiously with multiple stages of surgery in order to reduce the chance of amputation or osteomyelitis. Using a temporary dorsal plate for maintaining the reduction of the talonavicular joint for 2 to 3 months is recommended.

## Conflict of interest:

 None declared.

## References

[1] Dhillon MS, Bali K, Prabhakar S (2011). Controversies in calcaneus fracture management: a systematic review of the literature. Musculoskelet Surg.

[2] Haapasalo H, Laine HJ, Mäenpää H, Wretenberg P, Kannus P, Mattila VM (2017). Epidemiology of calcaneal fractures in Finland. Foot Ankle Surg.

[3] Ricci WM, Bellabarba C, Sanders R (2002). Transcalcaneal talonavicular dislocation. J Bone Joint Surg Am.

[4] Coltart WD (1952). Aviator's astragalus. J Bone Joint Surg Br.

[5] Kleiger B (1976). Injuries of the talus and its joints. Clin Orthop Relat Res.

[6] Ebraheim NA, Savolaine ER, Paley K, Jackson WT (1993). Comminuted fracture of the calcaneus associated with subluxation of the talus. Foot Ankle.

[7] Lwin CT, Garde A, Ribbans W (2007). Closed transcalcaneal fracture with talonavicular subluxation. Foot and ankle surgery.

[8] Galanakos SP, Papathanasiou V, Sofianos IP (2011). Transcalcaneal talonavicular dislocation associated with an open comminuted calcaneal fracture: a case report. Clin Podiatr Med Surg.

[9] Ventham NT, Phadnis J, Sujenthiran A, Trompeter AJ, Ramesh P (2013). Isolated transcalcaneal talonavicular dislocation: a severe injury related to a low-energy mechanism. The Journal of Foot and Ankle Surgery.

[10] Firoozabadi R, Kramer PA, Benirschke SK (2013). Plantar medial wounds associated with calcaneal fractures. Foot Ankle Int.

